# Association between thyroid disorders and oral health: OHRQoL, KAP and self-rated oral health assessment-A structural equation modelling approach

**DOI:** 10.6026/973206300220559

**Published:** 2026-01-31

**Authors:** Yamini Rajachandrasekaran, R. Riya Yazhini, Deepika Kottai Gandhi, Sumathi H Rao, Shifa Fathima Shajahan, Geetha Thirugnanasambandham

**Affiliations:** 1 Department of Periodontics, Sathyabama Dental College and Hospital, Chennai, Tamilnadu India; 2 Department of Endodontics and Conservative Dentistry, C.S.I College of Dental Sciences and Research, Madurai, Tamilnadu, India

**Keywords:** Quality of life, self-report, structural equation modelling, health knowledge, attitudes, practice, thyroid diseases, oral health

## Abstract

Thyroid disorders influence oral health through metabolic, immune, and behavioural changes, affecting oral health-related quality of life (OHRQoL). This cross-sectional study among 254 patients with thyroid disorders in Tamil Nadu evaluated OHRQoL, knowledge, attitude, practices (KAP), and self-rated oral health (SROH) using validated Tamil tools. Participants exhibited moderate knowledge and practices, positive attitudes, and moderate OHRQoL impairment. Knowledge showed positive correlations with attitude and practice but a negative relationship with SROH. Regression analysis identified knowledge, thyroid type, and disease duration as significant predictors of OHRQoL. Structural equation modelling confirmed acceptable model fit (χ^2^/df=3.289; RMSEA=0.095; CFI=0.907) and revealed interrelations among behavioural, perceptual, and clinical domains. Integrating oral health education and routine dental screening into endocrine care may improve oral and systemic health outcomes in this population.

## Background:

Thyroid disorders are among the most common endocrine disturbances globally, affecting approximately 5-10% of the population, with a higher prevalence among women and the elderly [1]. Thyroid diseases range from functional disorders such as hypo- and hyperthyroidism to autoimmune forms, including Hashimoto's thyroiditis and Graves' disease. Despite its small size, the thyroid gland is vital for metabolism, growth, and immune regulation through the release of thyroxine (T4) and triiodothyronine (T3) [2]. Recent evidence has pointed toward a bidirectional relationship between systemic endocrine imbalances and oral health, particularly periodontal disease [3]. Thyroid dysfunction can alter the host immune response, elevate pro-inflammatory markers, and affect the bone remodelling process, all of which may predispose individuals to periodontal diseases [4]. In addition, thyroid conditions have been associated with delayed wound healing, xerostomia, and increased susceptibility to oral infections [5]. Specific oral symptoms vary depending on the type of thyroid dysfunction. In hypothyroidism, common manifestations include macroglossia, delayed tooth eruption, salivary gland enlargement, oral burning sensations (clinically termed as burning mouth syndrome), and an elevated likelihood of periodontal disease due to impaired healing and reduced metabolic activity [6]. Conversely, hyperthyroidism may present with accelerated dental eruption in children, increased susceptibility to caries and periodontitis, tremors that affect oral hygiene practices, and, in some cases, osteoporosis of the jaws, which can compromise tooth stability and implant success [7]. Both conditions are also linked to dry mouth (xerostomia) and taste alterations, which further diminish "Oral health-related quality of life (QHRQoL)" [8]. These oral modifications can significantly influence an individual's daily functioning, overall well-being, and personal perception of their oral health. OHRQoL offers insight into how oral health touches different aspects of living, from physical comfort to emotional health and social participation [9]. In thyroid patients, deteriorated oral health status can further compound existing quality-of-life issues, making OHRQoL a valuable tool in evaluating disease impact. Evaluating "Knowledge, Attitude, and Practice (KAP)" linked to oral health enables the identification of behavioural determinants of hygiene habits and healthcare use in individuals with thyroid dysfunction [10]. Furthermore, self-rated oral condition has been considered to be a reliable predictor of clinical outcomes and a useful subjective measure of perceived health needs [11]. Despite the known associations, integrated models that evaluate the interplay between thyroid dysfunction, OHRQoL, KAP, and self-rated oral health are scarce. A more comprehensive understanding of these relationships requires robust statistical tools that can accommodate complex variable interdependencies. Structural Equation Modelling (SEM) is an advanced statistical method that enables concurrent evaluation of relationships between both observed and latent constructs [12, 13]. It helps in mapping direct and indirect influences on health outcomes, making it well-suited for analysing complex behaviours and perceptions in chronic illnesses. Therefore, it is of interest to evaluate the knowledge, attitudes, practices, oral health-related quality of life (OHRQoL), and self-rated oral health of individuals with thyroid disorders, and to explore the interrelationships among these factors using a structural equation modeling framework.

## Materials and Methods:

## Study design and population:

A cross sectional study was carried out over six months at a Primary Health Centre situated in Ramanathapuram (Ramnad) district, Tamil Nadu. Ethical clearance for the project was granted by the Institutional Review Board (Ref 486/IRB-IBSEC/SIST dated 10th December 2024). The number of participants was estimated in advance using Soper's a priori sample size calculator, which is frequently applied in research that uses Structural Equation Modelling (SEM). By considering a model composed of five underlying factors and forty measured indicators, the program suggested that about 200 subjects would be needed to obtain a statistical power of 0.80 at a 5% level of significance. To ensure adequate power in case of non-response or incomplete data, the target was increased to 250 participants, thereby strengthening the reliability of later SEM interpretations. Individuals were enrolled only after providing written consent. Eligible participants were adults aged 18 years and above who had a confirmed medical diagnosis of hypothyroidism or hyperthyroidism, could read and understand Tamil, and agreed voluntarily to take part in the study. People with systemic illnesses unrelated to thyroid function, and women who were pregnant or lactating, were not included so that oral health outcomes would not be influenced by unrelated medical factors.

## Data collection:

Information was obtained through face-to-face interviews using a structured questionnaire prepared in Tamil. The instrument had four parts. The opening section documented basic demographic and clinical information such as age, sex, type of thyroid disorder, and duration of diagnosis. The second portion employed the Tamil translation of the Oral Health Impact Profile-14 (OHIP-14) [14] to evaluate oral health-related quality of life (OHRQoL). Each statement was rated from 0 ("never") to 4 ("always") on a five-point scale to indicate how often participants experienced oral pain, functional difficulties, or emotional discomfort. The next portion contained a specially designed Knowledge, Attitude, and Practice (KAP) tool that explored how participants understood and managed the relationship between thyroid disease and oral health. The final section asked a single question on self-rated oral health: "How would you describe the present condition of your teeth and gums?" This single-item measure captured participants' overall perception of their oral condition in a simple and comprehensible format.

## Questionnaire development:

The Tamil OHIP-14 used in this study had previously been verified for cultural and linguistic relevance by Rishikeshan (2022) [14]. The KAP and self-rating components were drafted and evaluated by a multidisciplinary expert panel of ten members, including dental professionals and general physicians. Every item was reviewed for precision, clarity, and relevance on a four-point scale, and the Face Validity Index (FVI) was computed following the approach outlined by Yusoff (2019) [15]. This validation ensured that the questionnaire was suitable and easily understandable for the local Tamil-speaking population. The questionnaire was administered to participants at the health centre. Responses were verified on site for completeness before data entry. All data were coded and organised using Microsoft Excel to facilitate cleaning and management. Subsequently, descriptive statistical procedures and Structural Equation Modelling (SEM) were carried out to explore the interconnections between OHRQoL, KAP scores, and self-rated oral health, following previously established analytical frameworks [12].

## Statistical analysis:

IBM SPSS Statistics for Windows, Version 25.0, created by IBM Corp. (Armonk, NY, USA), had applied for all statistical analyses, and IBM SPSS AMOS Version 25.0 was used for structural equation modeling (SEM). Statistical significance is described as a p-value of less than 0.05. Descriptive statistics described demographic details and major variables, including KAP, SROH, and OHRQoL. Cronbach's alpha usually assesses internal consistency of questionnaire, or associations between variables are examined through Pearson's correlation. Chi-square tests have been applied to examine association between thyroid type or KAP, SROH, and OHRQoL, while one-way ANOVA with Tukey's post hoc test has been utilized to compare differences across age categories or disease duration. We applied multiple linear regression to find out which factors most strongly predicted OHRQoL. SEM employed the maximum likelihood estimation method to assess direct and indirect pathways between latent constructs (knowledge, attitude, practice) and observed variables (SROH, OHRQoL, age, gender, thyroid type). Model fit was assessed through indices such as χ^2^/df, RMSEA, CFI, TLI, GFI, PCLOSE, and RMR, providing a comprehensive multivariate evaluation of the complex interrelationships.

## Results:

**Figure 1:**
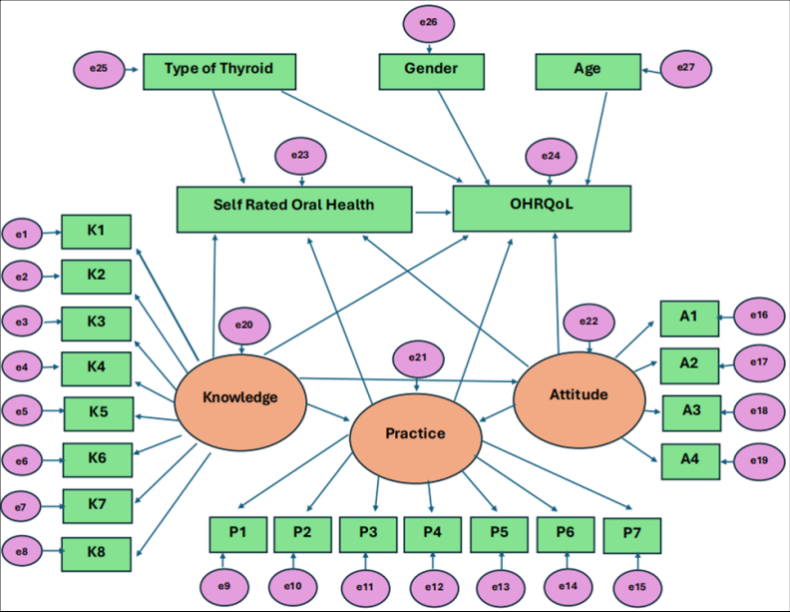
Conceptual framework illustrating the hypothesized relationships between thyroid-related factors, knowledge, attitude, practice (KAP), self-rated oral health (SROH), and oral health-related quality of life (OHRQoL) in individuals with thyroid disorders.

**Figure 2:**
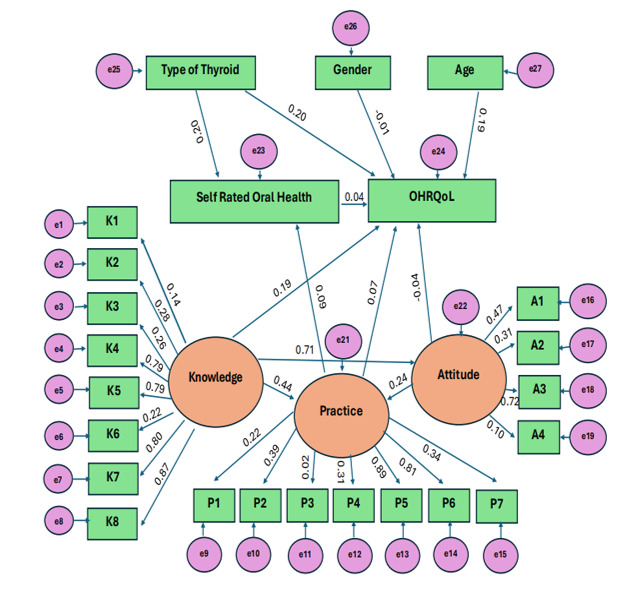
Structural Equation Model (SEM) depicting the relationships between knowledge, attitude, practice (KAP), self-rated oral health (SROH), oral health-related quality of life (OHRQoL), and demographic/clinical variables among patients with thyroid disorders. Standardized regression coefficients are shown along the paths.

The study included a total of 254 participants diagnosed with thyroid disorders, with a predominance of females (94.1%) and most belonging to the 18-30 years (32.3%) and 46-60 years (31.5%) age groups. The majority had hypothyroidism (66.9%) and had been diagnosed for 1-5 years (51.2%). The reliability analysis indicated acceptable to good internal consistency across the constructs: α = 0.717 for knowledge, α = 0.739 for attitude, α = 0.666 for practice (questionable), and α = 0.839 for the OHRQoL scale (Table 1). Participants demonstrated moderate oral health knowledge (mean = 3.48/8), positive attitudes (mean = 2.96/4), and moderate practices (mean = 4.84/7). Self-rated oral health was high (mean = 3.75/4), yet OHRQOL scores reflected a moderate impact (mean = 21.73/56) (Table 2). Many reported pain, discomfort, and psychological impacts, and 88% of respondents said their oral health was poor. Correlation analysis displayed that knowledge have a strong positive link with attitude (r = 0.435), practice (r = 0.458), and OHRQoL (r = 0.144), while it was negatively linked with self-rated oral health (r = -0.124) (Table 3). Chi-square tests indicated significant links between thyroid type and all key variables (Table 4). ANOVA further showed that both age and the length of disease having a notable impact on OHRQoL (p < 0.001 and p =0.009, respectively). Participants diagnosed within the past year reported better OHRQoL compared with those living with the condition for 6-10 years (p = 0.006). Multiple linear regression revealed that knowledge (β=0.204, p=0.006), thyroid type (β=0.279, p<0.001), or the duration of thyroid disorder (β=0.127, p=0.043) have been significant predictors of OHRQoL (Table 5), with no multicollinearity observed (VIFs < 2). To further explore interrelationships, AMOS software was employed to conduct the Structural Equation Modelling (SEM). The hypothesized model included latent constructs for Knowledge, Attitude, and Practice, and observed variables for SROH, OHRQoL, Age, Gender, and Thyroid Type (Figure 1). The model demonstrated an acceptable to marginal fit, with χ^2^/df = 3.289, RMSEA = 0.095 (indicating marginal fit), CFI = 0.907, TLI = 0.946, GFI = 0.848, PCLOSE = 0.06, and RMR = 0.017 (Table 6). Structural Equation Modelling (SEM) highlighted meaningful pathways: knowledge exerted a strong direct effect on attitude (β=0.715, p=0.036) or an indirect influence on practice (β=0.440, p=0.050). In turn, attitude showed a direct positive effect on practice (β=0.249, p=0.016). Attitude has been found to had a significant negative indirect effect on SROH (β=-0.184, p=0.024). In contrast, knowledge (β=-0.033, p=0.705) or practice (β=0.098, p=0.299) did not emerge as significant predictors of SROH. In relation to OHRQoL, all four factors demonstrated a significant impact: knowledge (β=0.190, p=0.050), attitude (β=-0.040, p=0.030), practice (β=0.070, p=0.041), and SROH (β=0.048, p=0.046) (Table 7). Demographic and clinical factors played a more substantial role-Thyroid Type significantly predicted both OHRQoL (β=0.209, p<0.001) and SROH (β=0.205, p=0.002), while Age also significantly influenced OHRQoL (β=0.195, p=0.002); Gender showed no effect (β = -0.017, p = 0.777). The SEM path diagram supported these findings and illustrated the tested model and its associations (Figure 2).

## Discussion:

This study examined the interplay between OHRQoL, knowledge, attitudes, or practices (KAP), and SROH among individuals with thyroid dysfunction. The findings underscore the multidimensional burden of thyroid disorders, which extends beyond systemic symptoms to significantly affect oral health perceptions, behaviours, or overall quality of life. Consistent with previous literature, hypothyroidism was more prevalent than hyperthyroidism in our sample and more common among females, particularly in the middle-aged group, mirroring well-documented global and European epidemiological patterns [1, 5, 16]. The scales used in the study demonstrated acceptable internal consistency, particularly for the OHIP-14, knowledge, and attitude domains. However, the practice domain showed questionable reliability (α = 0.666), which, while marginally acceptable, could be attributed to varied interpretation of behavioural items, self-report bias, or the limited availability of dental services-a trend also reported in KAP validation studies among chronic disease and rural populations [10, 17, 18]. Despite demonstrating moderate oral health knowledge and practices, participants exhibited relatively positive attitudes. This discrepancy, where attitudes are stronger than actual behaviours, has been described in health behaviour models and may reflect barriers like cost, time, or disease-related fatigue that inhibit the translation of intent into practice [19, 20].

Interestingly, although participants rated their oral health highly on a single-item scale (mean SROH = 3.75/4), 88% also reported poor oral health when asked globally, and their OHIP-14 scores revealed moderate impairment (mean = 21.73/56). This mismatch suggests a perceptual gap, where patients normalize symptoms or develop adaptive coping mechanisms, a phenomenon similarly noted in patients with other chronic diseases [11, 21]. Correlation analysis reinforced the conceptual framework of the KAP model, revealing strong links between knowledge, attitude, and practice. These finding highlights that gaining awareness is often an important first step toward influencing behaviour [10, 12]. Interestingly, knowledge showed a negative correlation with SROH (r = -0.124), suggesting that individuals with higher awareness may judge their oral health more critically. This "inverse awareness effect" has also been noted in self-management research, where better-informed patients often become more attuned to recognizing health shortcomings [11, 20]. Our analysis revealed significant associations between the type of thyroid disorder and all major variables. Hypothyroidism is commonly linked to macroglossia, xerostomia, burning mouth, and impaired wound healing, while hyperthyroidism often results in tremors affecting brushing, increased metabolic turnover, and greater susceptibility to caries and periodontal disease-all of which were reflected in patient responses [4, 5, 6, 7-8]. Disease duration also significantly affected OHRQoL, with longer-standing conditions correlating with greater perceived burden, supporting studies suggesting cumulative systemic and oral complications over time in chronic illnesses [3, 21].

Multiple regressions showed that knowledge, thyroid type, and disease duration were significant predictors of OHRQoL, reaffirming the combined contribution of behavioural as well as clinical factors in determining oral health consequences. This finding aligns with the biological framework linking thyroid function to oral health, where hormonal imbalances, immune disturbances, and inflammatory processes play a role in development of oral conditions [4, 5]. Structural Equation Modelling (SEM) added deeper insight by simultaneously analysing multiple pathways. The model demonstrated acceptable fit (χ^2^/df = 3.289; RMSEA = 0.095; CFI = 0.907; TLI = 0.946), supporting its structural validity [12, 13]. Strong direct effects of knowledge on attitude (β = 0.715) and indirect effects on practice (β = 0.440) were found, which is consistent with well-established theories of health behavior, including the Theory of Planned Behavior and the Health Belief Model [22]. Attitude had a significant positive effect on practice (β = 0.249) but was also linked to a negative indirect impact on SROH (β = -0.184), suggesting that individuals with higher oral health concerns may rate their own health more critically. This aligns with findings from health psychology, where greater awareness can lower perceived well-being even when objective health status is stable [11, 20]. Interestingly, neither knowledge nor practice had a direct effect on SROH, possibly due to optimism bias or ceiling effects in self-rating scales [11]. This further emphasizes the subjective nature of SROH compared to standardized OHRQoL tools like OHIP-14. OHRQoL was significantly predicted by knowledge (β = 0.190), attitude (β = -0.040), practice (β = 0.070), and SROH (β = 0.048), confirming that both behavioural and perceptual factors impact quality of life related to oral health. These results echo multidimensional model of OHRQoL proposed by Sischo and Broder, which integrates physical, psychological, and behavioural domains [9]. Among demographic and clinical factors, thyroid type and age significantly predicted OHRQoL, with older participants reporting worse outcomes, consistent with studies on aging and oral health deterioration [3, 23, 24]. Gender, however, did not significantly affect outcomes in our model, which may be due to the female-dominated sample or culturally mediated reporting bias-a factor warranting further exploration. Overall, these findings emphasize the need to promote oral health awareness and surveillance within endocrine care pathways. Tailored interventions should not only improve general knowledge but also address thyroid-specific oral manifestations and encourage practical behavioural change. Routine oral screenings, patient counselling, and interprofessional collaboration between dentists and endocrinologists could bridge current care gaps. Additionally, motivational strategies such as behaviour modelling, self-monitoring, and personalized oral hygiene plans may help overcome the attitude-practice gap observed within this population. Further investigations should explore longitudinal patterns in OHRQoL and incorporate objective clinical indices to strengthen causal inference and triangulate self-reported data.

## Conclusion:

We show that thyroid disorders can adversely affect QHRQoL, with both behavioral elements (knowledge, attitude, or practice) and clinical variables (thyroid type, age, and duration of the condition) contributing significantly. Structural equation modelling confirmed the pathways linking these determinants, underscoring the complex interplay between systemic disease and oral health. Integrating oral-health promotion into thyroid management can therefore improve overall patient well-being.

## Figures and Tables

**Table 1:** Internal consistency reliability (Cronbach's Alpha) of study constructs

**Construct**	**Number of Items**	**Cronbach's Alpha (α)**	**Interpretation**
Knowledge	8	0.717	Acceptable reliability
Attitude	4	0.739	Acceptable reliability
Practice	7	0.666	Questionable reliability
OHRQoL	14	0.839	Good reliability
SROH	1	Not Applicable	Single-item measure

**Table 2:** Descriptive statistics for main variables

**Variable**	**Mean**	**SD**	**Min**	**Max**
Knowledge Total	3.48	1.74	0	8
Attitude Total	2.96	0.75	1	4
Practice Total	4.84	1.67	1	7
Self-rated Oral Health	3.75	0.683	1	4
OHRQoL Total	21.73	6.356	14	41

**Table 3:** Pearson correlation matrix of primary study variables

**Variables**	**Knowledge**	**Practisce**	**Attitude**	**OHRQoL**	**Self-Rated Oral Health (SROH)**
Knowledge	1	.458**	.435**	.144*	-.124*
Practice		1	.207**	0.041	-.066
Attitude			1	0.042	-.113
OHRQoL (OHIP Total)				1	0.044
Self-Rated Oral Health					1

**Table 4:** Association between type of thyroid disorder and KAP, SROH, and OHRQOL

**Types Of Thyroid**	**Variable**	**χ^2^ (df)**	**p-value**
Hypothyroid	Knowledge Score	23.306 (8)	0.003
and	Practice Score	37.691 (6)	0
Hyperthyroid	Attitude Score	14.896 (3)	0.002
	OHRQOL Score	80.200 (22)	0
	SROH	11.344 (2)	0.003

**Table 5:** Multiple linear regression predicting oral health-related qualities of life (OHRQoL)

**Predictor Variable**	**B**	**SE B**	**β (Standardized)**	**t**	**p-value**	**VIF**
(Constant)	11.393	3.162	-	3.604	0	-
Knowledge Total	0.742	0.266	0.204	2.793	0.006	1.512
Practice Total	0.09	0.258	0.024	0.348	0.728	1.315
Attitude Total	-0.097	0.556	-0.012	-0.175	0.861	1.244
Self-Rated Oral Health (Q34)	0.054	0.568	0.006	0.095	0.925	1.059
Type of Thyroid	3.759	0.885	0.279	4.247	0	1.224
Duration of Thyroid Disease	1.158	0.569	0.127	2.035	0.043	1.102

**Table 6:** Model fit indices for the SEM framework

**Fit Index**	**Value**	**Recommended Threshold**	**Interpretation**
Chi-square/df	3.289	< 3	Acceptable
RMSEA	0.095	< 0.08	Marginal Fit
CFI	0.907	≥0.90	Acceptable
TLI	0.946	≥0.90	Acceptable
GFI	0.848	≥0.90	Moderate fit
PCLOSE	0.06	> 0.05	close-fitting
RMR	0.017	Close to 0	Acceptable

**Table 7:** Standardized regression weights indicating direct and potential indirect effects in the SEM model

**Path**	**Standardized direct Effect**	**p-value**	**Standardized Indirect Effect**	**p-value**
Attitude ← Knowledge	0.715	0.036	-	-
Practice ← Knowledge	-	-	0.44	0.05
Practice ← Attitude	0.249	0.016	-	--
Self-rated oral health ← Knowledge	-	-	-0.033	0.705
Self-rated oral health ← Attitude	-	-	-0.184	0.024
Self-rated oral health ← Practice	0.098	0.299	-	-
OHRQoL ← Knowledge	0.19	0.05	-	-
OHRQoL ← Practice	0.07	0.041	-	-
OHRQoL ← Attitude	-0.040	0.03	-	-
OHRQoL ← Self rated oral health	0.048	0.046	-	-
OHRQoL ← Type of Thyroid	0.209	0	-	-
OHRQoL ← Age	0.195	0.002	-	-
OHRQoL ← Gender	-0.017	0.777	-	-
Self-rated oral health←Type of Thyroid	0.2050.002	-	-
